# *Vibrio cholerae*—An emerging pathogen in Austrian bathing waters?

**DOI:** 10.1007/s00508-023-02241-0

**Published:** 2023-08-02

**Authors:** Carmen Rehm, Claudia Kolm, Sonja Pleininger, Florian Heger, Alexander Indra, Georg H. Reischer, Andreas A. H. Farnleitner, Alexander K. T. Kirschner

**Affiliations:** 1 Division Water Quality and Health, Karl-Landsteiner University of Health Sciences, Krems, Austria; 2 Institute for Hygiene and Applied Immunology – Water Microbiology, Medical University Vienna, Vienna, Austria; 3 Interuniversity Cooperation Centre Water & Health,; 4 Institute for Chemical, Environmental and Bioscience Engineering, Research Group Microbiology and Molecular Diagnostics 166/5/3, Technische Universität Wien, Vienna, Austria; 5 Institute for Medical Microbiology and Hygiene, National Reference Centre for Vibrio cholerae, Austrian Agency for Health and Food Safety (AGES), Vienna, Austria; 6 Paracelsus Medical University Salzburg, Salzburg, Austria

**Keywords:** *Vibrio cholerae* non-O1/non-O139, Epidemiology, Environmental reservoirs, Climate change, Diagnostic tools

## Abstract

*Vibrio cholerae*, an important human pathogen, is naturally occurring in specific aquatic ecosystems. With very few exceptions, only the cholera-toxigenic strains belonging to the serogroups O1 and O139 are responsible for severe cholera outbreaks with epidemic or pandemic potential. All other nontoxigenic, non-O1/non-O139 *V. cholerae* (NTVC) strains may cause various other diseases, such as mild to severe infections of the ears, of the gastrointestinal and urinary tracts as well as wound and bloodstream infections. Older, immunocompromised people and patients with specific preconditions have an elevated risk. In recent years, worldwide reports demonstrated that NTVC infections are on the rise, caused amongst others by elevated water temperatures due to global warming.

The aim of this review is to summarize the knowledge gained during the past two decades on *V. cholerae* infections and its occurrence in bathing waters in Austria, with a special focus on the lake Neusiedler See. We investigated whether NTVC infections have increased and which specific environmental conditions favor the occurrence of NTVC. We present an overview of state of the art methods that are currently available for clinical and environmental diagnostics. A preliminary public health risk assessment concerning NTVC infections related to the Neusiedler See was established. In order to raise awareness of healthcare professionals for NTVC infections, typical symptoms, possible treatment options and the antibiotic resistance status of Austrian NTVC isolates are discussed.

## Introduction

*Vibrio cholerae* is a natural inhabitant of aquatic ecosystems and is known as one of the most important waterborne human pathogens [1]. The bacterium was originally discovered by Filippo Pacini in 1854 and confirmed 30 years later by Robert Koch, who first was able to grow a pure culture [2]. It was described as curved, comma-shaped and highly movable due to its polar flagellum [3]. This gram-negative bacterial species consists of a large number of genetically and phenotypically different strains and biotypes, which are further classified into more than 200 serogroups, based on the lipopolysaccharide O‑antigen [4]. Its full genome consisting of two chromosomes was first sequenced in the year 2000 [5].

### The disease cholera

So far, only strains belonging to the serogroups O1 and O139 (with a few exceptions [6, 7]) carry the pathogenicity factors *ctx* and *tcp* required to cause epidemic or even pandemic cholera outbreaks [1]. *Ctx* encodes for the cholera toxin, responsible for causing the devastating diarrheal disease cholera and is encoded on the filamentous phage ctxPhi [8]. The factor *tcp* is located in the *Vibrio* pathogenicity island (vpi1) and codes for the toxin co-regulated pilus. This pilus acts as an adhesion factor for ctxPhi carrying the cholera toxin gene and also facilitates the colonization of the epithelial surface in the intestine [9]. Cholera infections are characterized by a typical rice-water stool and a severe loss of fluids of up to 20 l per day with a high mortality of up to 50% if untreated, due to severe dehydration [10]. Treatment with appropriate rehydration medium [11, 12] as part of an effective case management reduces the mortality to less than 1% [13]. The primary infection route is via ingestion of (drinking) water resources with naturally occurring, endemic *V. cholerae* O1/O139. The further spread of the disease occurs via contaminated water or food as well as via person to person contact, primarily in areas lacking safe water supplies (fecal-oral transmission route) [14]. In addition to control water and sanitation, vaccines are used for prevention in areas that are at high risk for cholera infections [15]. According to the World Health Organization (WHO), researchers have estimated that each year there are 1.3–4.0 million cases of cholera, and 21,000–143,000 deaths worldwide [12, 16].

Major ongoing cholera outbreaks have been reported from several countries in Africa and Asia in 2022 [17]. In contrast, cholera infections hardly ever occur in Europe and in all cases there is a connection to travelling to cholera endemic areas. In 2022, there were three documented cases in Europe [18], whilst the latest cases in Austria were in 2013, 2018 and 2022 which were reported to the ECDC via the European Surveillance System (TESSy). The exception to travel-related cholera was an accidental laboratory-induced cholera infection of a microbiology student in Austria in 2008 [19]. In general, tourists are at very low risk of infection because certain factors, such as the socioeconomic status influencing nutrition and gut microbiome and the type of travel, play an important role in the manifestation of the disease [14]. The risk of transmission within European countries is extremely low due to high sanitation and hygiene standards.

### Nontoxigenic *Vibrio cholerae* (NTVC) as a pathogenic agent

In contrast to cholera-toxigenic *V. cholerae* O1/O139, nontoxigenic, non-O1/non-O139 strains are present worldwide in specific aquatic environments and are able to cause different types of infections [20].

#### Pathogenicity and clinical manifestation

Nontoxigenic *Vibrio cholerae* (NTVC) strains possess a variety of pathogenicity factors (Table 1), enabling the pathogen to cause a wide range of diseases [21]. In addition to gastrointestinal infections, infections of ears, wounds and bloodstream (bacteremia, septicemia) are the most frequently reported [20, 22, 23]. Sporadic cases of necrotizing fasciitis [24], meningitis [25], and keratitis [26] have also been reported. Patients suffering from a gastrointestinal infections may have abdominal cramps, nausea and fever in addition to the main symptom diarrhea [27]. Ear infections are mostly manifested as otitis externa [28], or even as chronic otitis media [29]. Due to the high growth rate of the pathogen, wound infections can spread rapidly and lead to necrosis of the surrounding tissue (e.g. in the case of necrotizing fasciitis [24]) or septicemia with multiple organ failure [30], specifically in predisposed or immunocompromised individuals [23, 31]. In the majority of cases, the clinical presentation in healthy individuals is mild. Especially very young or older, immunocompromised patients or patients with chronic diseases (such as diabetes mellitus, hepatic disease, renal insufficiency, cardiovascular diseases) are at increased risk [23, 31].

**Table 1:** Overview of the most important pathogenicity factors found in *V.* *cholerae*

Pathogenicity factor	Abbreviation	Mechanism of pathogenicity	Source
Accessory cholera enteroxin	Ace	Increase of transcellular ion transport trough activation of chloride channels resulting in increase of fluid secretion (diarrhea)	[94]
Choleratoxin subunit a	ctxA	Cholera toxin consists of two subunits (subunit a is combined with max. five subunits b). Subunit a is responsible for intracellular toxicity resulting in massive diarrhea and dehydration	[95]
Choleratoxin subunit b	ctxB	Subunit b binds to a cell surface receptor, which facilitates the transmembrane transport leading to subsequent insertion of the toxin	[95]
Zonula occludens toxin	Zot	Decrease of intestinal tissue resistance/increase of intestinal permeability due to modification of intercellular tight junctions	[96]
Toxin-coregulated pilus	tcpA	Adhesion factor for the phage carrying the cholera toxin gene. Facilitation of the colonization of the epithelial surface in the intestine	[8]
Cholix toxin A	chxA	Required for infection of eukaryotes by receptor-mediated endocytosis, translocation to the host cytoplasm, and inhibition of protein synthesis	[97]
Hemolysin A	hlyA	Responsible for lysis of red blood cells	[98]
Motility-associated killing factor A	makA	Pore-forming toxin that promotes endocytosis in host cells	[99]
Mannose-sensitive hemagglutinin	mshA	Adhesion factor for attachment of *V. cholerae* to zooplankton and biofilm formation	[100]
Outer membrane protein U	ompU	Adhesion factor involved in cholera pathogenesis	[101]
Outer membrane protein T	ompT	Pore-forming toxin that provides transport of hydrophilic solutes through the outer membrane	[102]
Repeats in toxin A	rtxA	Plays an important role in cellular rounding and depolymerization of the actin cytoskeleton in host cells	[103]
Heat stable enterotoxin	Stn/sto	Causing severe diarrhea comparable to clinical cholera symptoms	[104]
Toxin regulator R	toxR	Activation of transcription of several virulence genes (e.g. toxT, ompU, ompT)	[105]
Toxin regulator T	toxT	Direct transcriptional activator of ctx and tcp	[106]
Type III secretion system	T3SS	Causes alteration of actin polymerization homeostasis, required for efficient intestinal colonization	[107]
Type VI secretion system	T6SS	Can translocate effector proteins into macrophages and covalently cross-link actin in vitro	[108]

Up to now, there is only limited information on the association of specific virulence factors with specific infection types. In a comparative study of German and Austrian isolates it was found that non-O1, non-O139 strains from patients with diarrheal symptoms possessed the type III secretions system (T3SS) and/or the multifunctional autoprocessing repeats-in-toxin (MARTX) toxin, which were not found in the strains associated with ear or wound infections [32]. Whether this is globally the case, remains to be investigated. Also, clinical isolates exhibit similar virulence gene profiles as environmental ones. It was thus concluded that many virulence traits of *V. cholerae* have evolved in response to biotic and abiotic pressure and serve adaptation purposes in the natural aquatic environment, at the same time as providing a prerequisite for infection of susceptible human hosts [21].

#### Treatment and antibiotic resistance

In most cases, diarrhea caused by NTVC is harmless and self-limiting. Generally, people with a gastrointestinal infection should recover within a few days [23]; however, antibiotic treatment is recommended for severe cases of diarrhea. Antibiotics are also the treatment of choice in cases of severe or prolonged ear infections, wound infections, septicemia or necrotizing fasciitis. Due to the high growth rate of the pathogen, rapid decisions based on antimicrobial susceptibility testing of the isolated pathogen are necessary to prevent severe outcomes, such as amputation of limbs or death, specifically with septicemia and necrotizing fasciitis. The first choice of antibiotics is doxycycline or ciprofloxacin (in case of gastrointestinal infections). For severe infections and septicemia, third generation cephalosporins should be added [33]. Previous studies on Austrian environmental isolates showed high proportions of antimicrobial resistance to sulfonamides (97.5%) and streptomycin (39%, [34]). Resistances to ampicillin were also observed in up to 21% of the investigated isolates. Clinical isolates from patients who were in contact with the same environments displayed similar resistance patterns, but some of them were additionally resistant to amoxicillin/clavulanic acid [34], the most widely used broad spectrum antibiotic. No resistances were found against all recommended first-line (see above) and last-line treatment options (4th generation cephalosporins, carbapenems). Similar results were observed in France, with resistances to streptomycin (22%), sulfonamides (44%) and ampicillin (9%) [35]. In German coastal samples of the North Sea and the Baltic Sea, some sporadic isolates with resistance to carbapenems, quinolones and folate pathway inhibitors were found [36]. Low resistance levels were also observed among 307 NTVC isolates from Chesapeake Bay, USA. Only 13% carried a phenotypic resistance to ampicillin or penicillin, one isolate was resistant to erythromycin [37]. Sulfonamides were only tested in combination with trimethoprim and did not show resistance for this combined treatment [37], as also observed in the Austrian and French studies [34, 35]. In contrast, in cholera epidemic countries, resistances to macrolides (erythromycin), fluoroquinolones (ciprofloxacin), trimethoprim/sulfamethoxazole and tetracycline have been reported with increasing trends for fluoroquinolones (ciprofloxacin, norfloxacin), quinolones (nalidixic acid) and aminoglycosides (gentamicin) [38]. Also, the presence of extended spectrum beta-lactamases (ESBLs) and carbapenemases were reported in some isolates [39]. Therefore, an antibiogram is absolutely mandatory for imported/travel-associated cases.

### Environmental reservoirs and transmission to humans

NTVC are natural inhabitants of marine and inland waters with mostly light to moderate salinity [20]. In countries with good sanitary and water hygiene conditions, NTVC thus do not enter a natural water body through human fecal pollution and there is no possibility of preventing their growth in suitable aquatic environments. Transmission to humans most frequently occurs via consumption of seafood or direct contact with water during recreation, such as bathing, swimming or water sports [20]. Besides salinity, different environmental parameters, such as organic and inorganic nutrients, pH, or biological interactions with plankton species are known to influence the occurrence of NTVC [40–42]. Specifically, *V. cholerae* has a high affinity for attachment to and colonization of chitin surfaces, such as zooplankton, which are considered as the main environmental reservoir [43]. The exoskeleton surface of a single colonized crustacean zooplankton individual has been shown to contain up to 10^4^ *V. cholerae* cells [44], which may have an impact on the likelihood of acquiring a gastrointestinal infection. In this way, zooplankton might act as “Trojan horses”, protecting *V. cholerae* during the gastric passage. The biofilm mode of life and interaction with zooplankton also enhances the natural competence for DNA uptake by transformation or horizontal gene transfer from neighboring *V. cholerae *cells and other bacterial species [45]. In this way, NTVCs obtain an evolutionary advantage by achieving a better adaptation to changing environments, including the acquisition of antibiotic resistance genes or new pathogenic traits [45]. Other environmental hosts of *V. cholerae* that may also act as potential transmission vehicles are egg masses of chironomids [46], fish [47] and waterfowl [48]. Interaction of *V. cholerae* was also reported with amoeba (such as *Acantamoeba castellanii *or* Tetrahymea pyriformis*), where the bacteria may escape digestion in food vacuoles and become hyperinfectious [49].

### Role of increasing water temperatures due to global warming

Temperature was identified as one of the main factors impacting the growth of environmental *V. cholerae *[20]. *V. cholerae* is very sensitive to cold temperatures (below 10–15 °C), where they enter the so-called VBNC (viable, but non-culturable) state, resulting in cell size reduction, reduced cell metabolism and cessation of cell division [50]. In the environment, an increase in temperature and the restoration of favorable conditions leads to a resuscitation of the VBNC cells, which also regain their normal phenotypic features [51].

On a global scale, temperatures have increased by approximately 1.1 °C since the late nineteenth century due to global warming [52]. For surface waters in specific regions, the average increase is even higher. For instance, the North Atlantic and North Sea experienced a 1.5 °C rise [53] while the Neusiedler See recorded a remarkable 1.9 °C increase in summer maximum water temperatures per decade over the past 50 years [54]. These observations indicate a high potential for an increase in summer concentrations and latitudinal expansion of suitable areas for *V. cholerae* [20, 55]. Ongoing warming of coastal regions and reduced salinity (caused by severe precipitation events) are expected to further support the spread of these bacteria on a global scale, especially in northern latitudes [20]. In fact, it has already been observed that plankton-associated *Vibrio* species (including *V. cholerae*) have significantly increased in their relative abundance in many oceans of the Northern Hemisphere, which is linked to increased sea-surface temperatures [53, 56, 57]. Consequently, *Vibrio *species were designated as barometers of climate change [58]. Higher water temperatures triggered by global warming were also linked to the observed increased numbers of infections with *V. cholerae* and other *Vibrio *species in the Baltic Sea, specifically during summer heat waves [59, 60]; however, to date no evidence exists whether the observed increased infection numbers were caused by higher *Vibrio* concentrations in the water or by a higher exposure of an increased number of recreating people. A considerably robust database (COVIS) providing evidence for increasing numbers of *V. cholerae* and other *Vibrio* infections is available for the USA [61]. For Europe or other regions in the world, no such databases exist.

### Epidemiology of NTVC in Austria

**Fig. 1:**
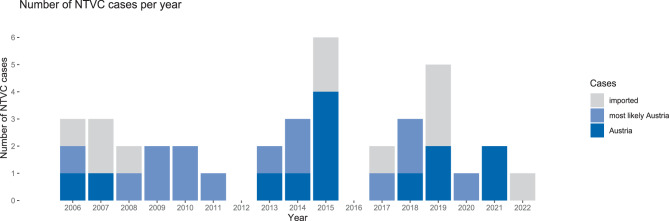
Reported cases of non-toxigenic *Vibrio cholerae* (NTVC) infections in Austria, from 2006 to 2022 (*n* = 38). *Dark blue bars* represent cases with a confirmed travel history to local bathing waters. *Medium blue bars* represent cases where information is insufficient but there is a likely association with local bathing waters. The *grey bars* are cases of travel-associated NTVC infections

**Fig. 2:**
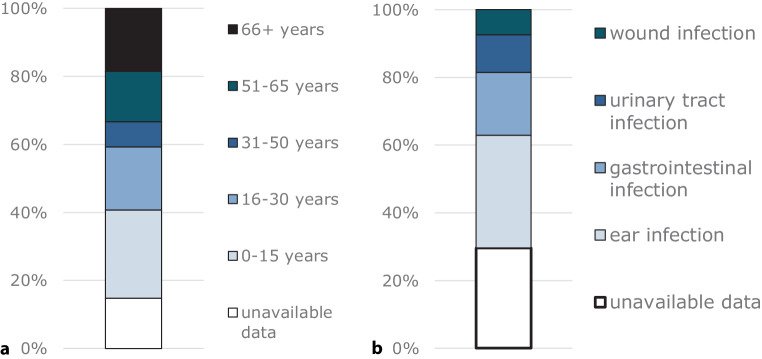
Age distribution (**a**) and type of infection (**b**) of the 27 non-toxigenic *Vibrio cholerae* (NTVC) cases reported between 2006 and 2022 with Austrian and most likely Austrian origin. Additional information: Sex: 53% male, 26% female, 21% insufficient data; Age median = 32 years, min. = 4 years, max. = 80 years

In recent years, several cases of NTVC infections have been documented in Austria with a local history, specifically associated with recreational activities at bathing sites. Before 2005, a total of 5 cases were reported with a definitive association with Lake Neusiedler See, a large subsaline shallow lake in Eastern Austria. Among these, three cases of otitis media, one case of otitis externa and one fatal case of septicemia were reported [28]. In addition, 5 cases of travel history abroad (all diarrhea) and 3 cases of unknown local travel history (all otitis media) were observed [28]. Since 2006, a total of 42 *V. cholerae* infections have been documented in Austria. Out of these cases, four cases were excluded from the dataset, which were identified as *V. cholerae *O1 infection. Thus, in total 27 NTVC infections that were most likely (*n* = 14) or likely (*n* = 13) acquired in Austria were reported, whilst the rest (*n* = 11) were imported infections from foreign countries (Fig. 1). In general, no increasing trend or any other pattern could be observed. In fact, most cases occurred in 2015, which was the second warmest year in this country since the beginning of records. During the summer heat wave in 2015, two severe cases of necrotizing fasciitis occurred at two bathing ponds south of Vienna (Eastern Austria), one with fatal outcome [24]. All other cases with a known travel history in Austria were associated with Lake Neusiedler See. Infections regarding the categories “Austria” and “most likely Austria” occurred in patients aged 4–80 years, while the majority of patients were between 4 and 30 years old (40%) or between 51 and 80 years old (29%); only 6% of the patients were between 31 and 50 years old, no age information was available for 25% of the patients (Fig. 2a). Regarding the type of infection, ear infections were the most frequent type, followed by gastrointestinal infections. Urinary tract infections and wound infections (necrotizing fasciitis) occurred two times each (Fig. 2b).

Beyond that, it is anticipated that a considerable number of NTVC infections remain unreported, due to the absence of mandatory reporting requirements. Infections are mostly either mild and self-limiting or they can be treated easily with broad spectrum antibiotics without the need for isolation of NTVC as the causative agent. In addition, physicians are often unaware of NTVC as potential pathogens and thus NTVC infections remain underdiagnosed. In contrast to other regions in the world (see above), no increase in cases has been observed during the past two decades, and severe manifestations of the disease (such as necrotizing fasciitis or septicemia) still appear to be a very rare phenomenon.

### Diagnostic methods for NTVC in clinical samples

**Fig. 3:**
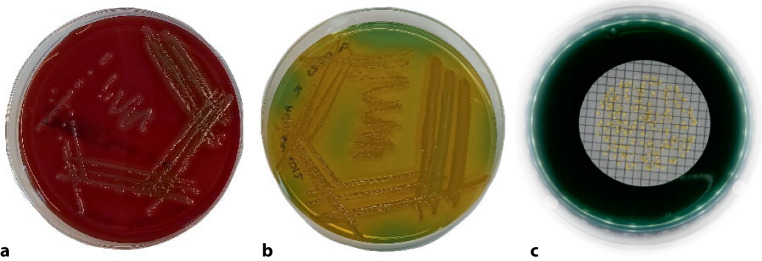
Typical appearance of non-toxigenic *Vibrio cholerae* (NTVC) on sheep-blood agar (**a**) and on thiosulfate citrate bile sucrose (TCBS) agar (**b**). Typical NTVC colonies on TCBS agar after filtration of 100 ml lake water sample through a 0.45 µm pore size nitrocellulose filter (**c**)

If an infection caused by NTVCs is suspected, patient samples are taken based on the clinical manifestation. Bacteria can be cultured on sheep blood agar and thiosulfate citrate bile sucrose (TCBS) agar from stool or blood samples, wound or ear swabs. The selective agar TCBS allows a suppression of most other bacteria and a differentiation of *Vibrio* species based on color of the colonies. Typical colonies (Fig. 3) have to be selected and confirmed by various identification methods such as matrix-assisted laser desorption/ionization-time of flight (MALDI-TOF) [62], analytical profile index (API) [63], multiplex PCR [64] or whole genome sequencing (WGS) [65]. While MALDI-TOF is a quick and cheap method, reliable identification is sometimes difficult, as differentiation from sister species such as *V. mimicus *[66], *V. metoecus* [67] and the recently described *V. paracholerae* [68] is hampered by incomplete data bases. In some data bases, *V. cholerae *isolates are indicated as *V. albensis*, which was formerly recognized as a separate species but was later integrated into *V. cholerae* (a non-O1/non-O139 serogroup [69]). As the NTVC populations show a very high genetic [70, 71] and phenotypic diversity [72], multiplex PCR and API methods can also be prone to misidentification when primer target sequences and metabolic properties show variations. The WGS is thus the most reliable method for species identification of NTVC, but comes with elevated costs. After identification of the isolate, simple serotyping with O1 and O139 agglutination sera is usually performed to quickly identify the toxigenic serogroups O1 and O139 [73]. Testing for other serogroups [4] is generally not performed in clinical practice. Furthermore, in severe or imported cases, an antibiogram has to be prepared to assess potential resistances [6]. Following the application of pulsed field gel electrophoresis (PFGE, [74]), ribotyping [75], amplified fragment length polymorphism (AFLP, [76]) and enterobacterial intergenic consensus sequence-PCR (ERIC-PCR, [77]) in the past three decades, current state of the art methods used for strain typing are multi-locus sequence typing (MLST) using a set of 7 housekeeping genes ([78], https://pubmlst.org/organisms/vibrio-cholerae) or core-genome MLST (cg-MLST) via WGS [79]. When applying WGS for identification and typing, pathogenicity factors and resistance genes can also be concomitantly identified.

### Diagnostic methods for NTVC in environmental samples

#### Cultivation

To assess the occurrence and abundance of NTVC in natural bathing waters, a cultivation-based standard most probable number approach adopted from the detection of this pathogen in foodstuffs is available [80]; however, this approach is highly elaborate and afflicted with a high degree of measurement uncertainty, specifically when quantitative results are needed. Thus, a membrane filtration approach for reliable quantification and a simple direct plating approach for rapid screening was recommended [64]. These approaches have been successfully applied for various bathing waters in Eastern Austria and Serbia [64, 81]. Briefly, for membrane filtration water samples are filtrated in different dilutions (e.g. from 100 ml to 0.1 ml) through 0.45 µm pore size nitrocellulose membrane filters and placed on selective agar plates (TCBS; Fig. 3). For direct plating, 500 µl water are directly streaked on predried TCBS agar plates. After incubation for 18–24 h at 37 °C, the typical presumptive colonies (~2 mm diameter, yellow, round shape) are counted and 5–10 representative colonies per sample are transferred to Columbia Agar without NaCl and incubated for another 24 h. Presumptive *V. cholerae *isolates grown on agar without NaCl may then be confirmed using multiplex-PCR or MALDI-TOF ([64]; see also above).

#### Cultivation independent methods

Alternative cultivation-independent methods that have been developed and applied in recent years are based on quantitative PCR (qPCR) and fluorescence microscopy in combination with solid-phase cytometry (SPC). In our laboratory, a triplex qPCR has been developed to simultaneously quantify toxigenic and non-toxigenic *V. cholerae* in environmental water samples [82, 83]. This method has been modified recently concerning sample concentration and DNA extraction and applied in Serbian lakes and ponds [81]. Briefly, 50–200 ml water samples are filtered onto 0.2 μm pore size polycarbonate filters, followed by phenol-chloroform DNA extraction [84]. Aliquots of the DNA extracts are used for the multiplex qPCR targeting both cholera-toxigenic (*ctxA*) and non-toxigenic *V. cholerae* (outer membrane protein W, ompW, a protein that is present in all *V. cholerae* strains [85]). In addition, an internal amplification control is included in the triplex qPCR assay to indicate potential PCR inhibition.

A highly sensitive microscopic quantification method was also developed, based on the specific staining of *V. cholerae* cells via fluorescence in-situ hybridization (FISH) combined with catalyzed reporter deposition (CARD) and the detection of the stained cells with a solid phase cytometer (SPC) combined with a fluorescence microscope. This staining technique is based on specific genetic probes that allow identifying and quantifying specific microorganisms with low ribosome content. In CARD-FISH, the RNA-targeted probe is labelled with horseradish peroxidase, to enzymatically increase weak fluorescence signals. In combination with SPC it was possible to quantify *V. cholerae* cells in the Lake Neusiedler See and its adjacent soda pools year-round, even at very low cell numbers in winter [42, 86]. A disadvantage of the method is that it is highly elaborate and can only be performed in specialized laboratories.

### NTVC in Eastern Austrian lakes and ponds

#### Lake Neusiedler See and adjacent soda pools as hotspots of NTVC

The occurrence of infections caused by NTVC related to recreational activities in Austria was first reported at the beginning of this century for Lake Neusiedler See [28]. Based on these reports, investigations were initiated to study the prevalence of NTVC in this lake. These cultivation-based qualitative investigations illustrated that culturable NTVC were present during the warmer seasons (from May to September) and that their growth was mainly dependent on the temperature and the amount and composition of organic matter [41]. Higher concentrations of humic substances, as they occur in the broad reed stand of the lake inhibited the growth of these pathogens. The limnological characteristics of Neusiedler See (high pH-value around 8.8, electrical conductivity around 2 mS/cm, high nutrient concentrations and high water temperatures above 28 °C at the water surface), offer ideal conditions for the bacteria. The bacteria can either grow in a planktonic state in the water or in biofilms on the surfaces of a specific Cladoceran zooplankton species, *Diaphanosoma mongolianum* [44]. With the developed cell-based method, cell counts of up to 7.7 × 10^5^ per zooplankton individual were observed [44].

**Fig. 4:**
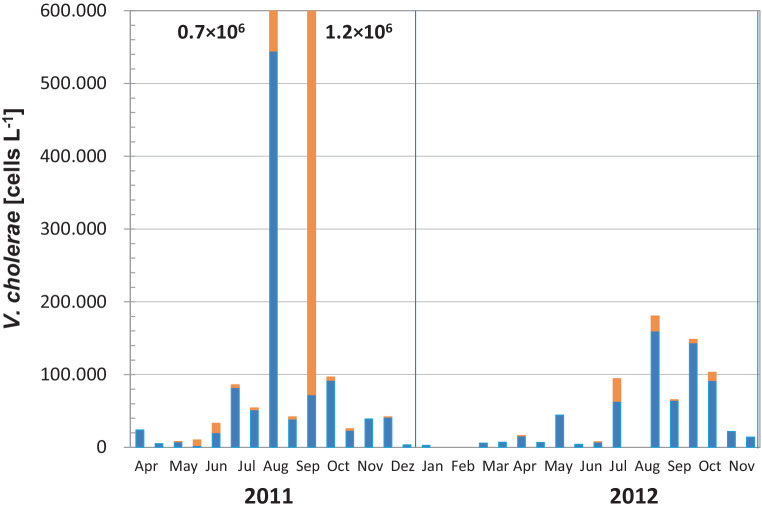
Seasonal abundance of *V.* *cholerae* cells in water (blue bars) and on zooplankton (orange bars) at a representative sampling site of Lake Neusiedler See (data taken from Schauer et al. [42])

During a 2-year seasonal study at the Neusiedler See and three adjacent small highly saline soda pools (*Salzlacken*) *V. cholerae* abundances up to 5.5 × 10^5^ cells L^−1^ were recorded in the lake water and up to 1.2 × 10^6^ cells L^−1^ on zooplankton (Fig. 4); however, the zooplankton was a relevant source of *V. cholerae* only during a short period in late summer. Also resuspended sediment can act as a source of *V. cholerae* in the water body; up to 10^4^ *V. cholerae* per g sediment were observed [42]. In the soda pools, numbers up to 56 × 10^6^ *V. cholerae* cells per L were recorded, representing (by tenfold) the highest concentrations observed so far for other ecosystems worldwide [42]. Interestingly, an infection with *V. cholerae* in Neusiedler See was also reported during the cold season (end of November), when no culturable *V. cholerae* are present [28]. With the developed CARD-FISH SPC method, *V. cholerae* cells could also be detected at low concentrations during the winter season, where they obviously survive in the viable but non-culturable (VBNC) state [42] and may regain their infectivity after resuscitation in a suitable host.

The diversity of the NTVC strains in Neusiedler See was assessed via multi-locus sequence analysis (MLSA). A total of 472 isolates were collected from water and zooplankton over a time span of 2 years, leading to the identification of 94 different sequence types [70]. The highest diversity was observed in the reed habitat with 71 unique phylogenetic lineages, whereas only 2 unique sequence types were found in the open water habitat. Interestingly, two clinical isolates from patients who were infected in 2008 and 2009, had identical sequences as strains isolated from the lake in 2011 and 2012, suggesting the long-term survival of specific clones within the lake. Even more interesting, a comparison with isolates from several European countries revealed that many NTVC isolates from Sweden, France, Romania and The Netherlands were genetically related to the strains present in the lake belonging to statistically supported monophyletic clades [70]. This phenomenon can be explained by the long-distance transfer of strains, likely facilitated by birds and/or humans. The Neusiedler See along with its adjacent soda pools therefore serve as bioreactors for the emergence of new strains with potentially new (pathogenic) properties.

#### Preliminary public health risk assessment for Lake Neusiedler See

Due to the facts that (i) no dose-response relationships for NTVC regarding intestinal and extraintestinal infections exist and that (ii) the responsible pathogenicity factors for these infections are not known, no state of the art quantitative risk assessment [87–89] can be performed. Thus, a preliminary risk assessment was performed for Lake Neusiedler See [42], based on the available NTVC abundance data, genotyping results from the multiplex PCR and reported minimum infectious doses for *V. cholerae* (oral exposition mode).

- Risk of acquiring cholera: during all years, not a single *ctx* and *tcp* positive *V. cholerae* O1/O139 isolate was found and not a single sample yielded a positive *ctx* signal by qPCR. Thus, the presence of any cholera-causing strains in Neusiedler See can be practically excluded.
- Risk of acquiring a gastrointestinal infection by NTVC: in the last 20 years, gastrointestinal infections (GI) caused by NTVC in connection with Neusiedler See were reported only rarely (5 cases documented). NTVC concentrations of 5.5 × 10^5^ cells per L lake water were observed. Adopting an infective dose of 10^5^–10^6^ cells from studies with healthy volunteers [90–92] one would have to swallow ∼200 ml–2 L of water to acquire a gastrointestinal infection. The presence of zooplankton might significantly lower the amount of water to be swallowed as these small animals can harbour up to 7.7 × 10^5^ *V. cholerae* cells per individual in specific periods of the year [44]. Thus, for susceptible individuals, a few zooplankton organisms may be sufficient for infection.
- Risk of acquiring a wound or ear infection by NTVC: there are no studies concerning the infective dose for ear or wound infections by NTVC, but it can be assumed that it may be only a few cells, based on exponential single-hit models for other pathogens [93]. Based on available epidemiological data, the number of severe infections is low and has only been reported three times for Austria [24]. Worldwide, severe infections have primarily been associated with vulnerable individuals with specific preconditions (such as immunodeficiency, cancer therapy, skin diseases, liver cirrhosis) placing them at an elevated risk. A significant number of unreported cases may be assumed in healthy individuals, as most of the ear or wound infections are self-limiting or easily treatable with broad spectrum antibiotics. To date, the Austrian NTVC strains do not harbor relevant antibiotic resistance traits against first-line and last-line antibiotics [34].

#### NTVC in other bathing waters in Eastern Austria

Due to the fact that in 2015, two extreme cases of necrotizing fasciitis were recorded in association with two ponds in Eastern Austria [24], a follow-up study to monitor the occurrence of NTVC in selected bathing ponds in Eastern Austria (Burgenland, Vienna, Lower Austria) was initiated. The NTVC abundance was monitored at 36 bathing sites by culture-based methods. Membrane filtration yielded the most reliable and sensitive results and allowed NTVC detection at 22 sites with concentrations up to 3.9 × 10^5^ CFU per L, all belonging to serogroups other than O1 and O139 and not coding for *ctx* or *tcp* [64]. This study showed that NTVC are more widespread in Eastern Austria than previously thought and that more detailed follow-up investigations are necessary to identify the key factors responsible for the occurrence of NTVC in Eastern Austrian bathing waters.

So far, NTVC was only detected in the most eastern Austrian lakes and ponds. In the more western lakes situated at mountainous or alpine altitudes, the occurrence of NTVC is unlikely, due to the different ecological conditions (lower conductivity, lower DOC content, lower summer temperatures in comparison to Eastern Austrian waters).

## Conclusion

NTVC infections are still rare in Austria. So far, reported domestic cases have only been linked to bathing waters in Eastern Austria, predominantly to Lake Neusiedler See. In this study, we did not find any evidence indicating a correlation between the increase in bathing water temperatures and a rise in reported NTVC infections over the past two decades. A specific situation was experienced in the heat wave summer of 2015, when a higher number of cases including two severe cases of necrotizing fasciitis (one with fatal outcome) were recorded. These cases were linked to two ponds in Lower Austria; however, in other heat wave summers (such as in 2003, 2012, 2017–2019) no such accumulation of cases was observed. Thus, despite the well-documented rise in *V. cholerae* infections due to global warming in other regions of the world (USA, Baltic Sea), such a scenario is still unclear for Austria.

Due to the high diversity of NTVC strains, various pathogenicity factors and varying host susceptibility, different kinds of infections can be caused by NTVC. As many of these infections proceed in a self-limiting way or are cured after the use of broad spectrum antibiotics, a substantial number of undiagnosed infections can be assumed. Generally, healthy individuals are not at risk of suffering or experiencing severe infections, but older persons with severe pre-existing illnesses and an immunocompromised status are at elevated risk and should be aware of this fact when recreating in waters with high NTVC concentrations. In cases of infections, due to the high growth rate of the pathogens, rapid antibiotic treatment is recommended, with first-line treatment options of ciprofloxacin, doxycycline and third-generation cephalosporins. In the case of a travel-associated NTVC infection, multidrug resistance is possible and an antibiogram should be conducted as soon as possible to guide appropriate treatment decisions.
